# Combination therapy: intermittent sorafenib with bevacizumab yields activity and decreased toxicity

**DOI:** 10.1038/sj.bjc.6605514

**Published:** 2010-01-05

**Authors:** J-M Lee, G A Sarosy, C M Annunziata, N Azad, L Minasian, H Kotz, J Squires, N Houston, E C Kohn

**Affiliations:** 1 Medical Ovarian Cancer Team, Medical Oncology Branch, Center for Cancer Research, National Cancer Institute, Bethesda, MD 20892, USA

**Keywords:** ovarian cancer, anti-angiogenesis, bevacizumab, sorafenib

## Abstract

**Background:**

We previously reported preliminary results of our phase I study of continuous daily sorafenib with bevacizumab every other week for solid tumours. Toxicity was moderate, leading to additional dose levels (DL) testing intermittent sorafenib dosing.

**Methods:**

Seventeen patients with advanced solid tumours were treated on three additional DLs testing sorafenib days 1–5 per week. Dose level 4 was sorafenib 200 mg twice daily (b.i.d.) and bevacizumab 5 mg kg^−1^. DL5 alternated between bevacizumab 10 mg kg^−1^-sorafenib 200 mg b.i.d. (A) and sorafenib 400 mg b.i.d. with bevacizumab 5 mg kg^−1^ (B). Outcome and toxicity data from 19 epithelial ovarian cancer (EOC) patients from DL 1–5 were analysed.

**Results:**

Fewer patients required sorafenib dose reduction with the intermittent schedule (41 *vs* 74% daily, *P*=0.01). Hand–foot skin reaction (HFSR) remained the primary cause of dose reduction (*n*=5). Partial responses (12%) or disease stabilisation ⩾4 months (53% median 6 (4–26)) occurred in most patients on the intermittent schedule. Partial response occurred in 47% EOC patients treated in pooled analysis of duration 4–37 months.

**Conclusion:**

Intermittent sorafenib dosing with bevacizumab has promising clinical activity and less sorafenib dose reduction and side effects, but does not ameliorate HFSR. We are conducting a phase II clinical trial with intermittent sorafenib and bevacizumab in patients with EOC.

Small-molecule signal-transduction inhibitors (STIs) with oral bioavailability have demonstrated single-agent clinical activity in tumours with documented molecular defects in dominant biochemical pathways. Signalling pathway targets inhibited by STIs include the bcr–abl fusion protein in chronic myelogenous leukaemia, and c-kit or epidermal growth factor receptor mutations in gastrointestinal stromal tumours and non–small-cell lung cancer (Druker *et al*, 2001; Demetri *et al*, 2002; Lynch *et al*, 2004). Combination strategies using signal inhibitory agents with related targets have the potential to induce biochemical and clinical synergism. Signalling interactions would lead to the expectation that therapeutic interruption of pathways in series (vertical inhibition) may allow administration of lower doses of agents that intersect the pathway at multiple sites (Araujo *et al*, 2007).

Sorafenib is a multikinase inhibitor that targets RAF kinase, VEGFR2, platelet-derived growth factor-*α* and -*β*, and c-KIT (Escudier *et al*, 2005; Llovet *et al*, 2008; Azad *et al*, 2008b). Sorafenib is approved for use in advanced renal cell carcinoma (RCC) and hepatocellular carcinoma (Escudier *et al*, 2007; Llovet *et al*, 2008). Bevacizumab, an anti-vascular endothelial growth factor (VEGF) antibody, demonstrated single-agent activity in relapsed epithelial ovarian cancer (EOC), with a response rate of 17% in platinum-resistant patients (Burger *et al*, 2007; Cannistra *et al*, 2007). The addition of bevacizumab to chemotherapy has resulted in improved survival in phase III studies in patients with metastatic colorectal, non-small-cell lung and breast cancers (Hurwitz *et al*, 2005; Sandler *et al*, 2006; Miller *et al*, 2007).

We reported preliminary results of a phase I trial of a combination of bevacizumab and sorafenib in 39 patients with a variety of tumour types (Azad *et al*, 2008a, 2008b). Our results showed an unexpectedly high partial response rate (PR=46%) in patients with relapsed EOC compared with 16–21% response rate reported with bevacizumab alone. The regimen of daily oral sorafenib 200 mg twice daily (b.i.d) and bevacizumab 5 mg kg^−1^ every 2 weeks was tolerable. However, 62% of patients experienced fatigue, hand–foot skin reaction (HFSR) syndrome, hypertension, proteinuria, and/or thrombocytopenia necessitating dose reduction to daily sorafenib after 2–4 months of therapy. In recognition of the interactive toxicity, we moved to an intermittent schedule using 5 days of sorafenib each week. We now provide results of intermittent sorafenib dosing on three dose levels (DLs) in 17 additional patients with solid tumours. We include a pooled analysis of outcome in the 19 relapsed EOC patients treated with bevacizumab and sorafenib in all administered DLs of the trial, both those receiving continuous sorafenib in the first part of the trial, as well as those receiving intermittent sorafenib in these three DLs.

## Patients and methods

### Eligibility

This study was approved by the Institutional Review Board of the National Cancer Institute. Written informed consent was obtained from all patients before enrolment. Eligibility requirements were as follows: advanced solid tumours, no treatment for at least 4 weeks, ECOG performance status of 0 or 1, leukocyte count ⩾3000 *μ*l^−1^, absolute neutrophil count >1200 *μ*l^−1^, platelet count ⩾100 000 *μ*l^−1^, serum creatinine ⩽1.5 mg per 100 ml, transaminases ⩽2.5 × upper limit of normal (ULN), bilirubin ⩽1.5 mg per 100 ml, and normal amylase and lipase. Coagulation parameters within 1.25 × ULN and (corrected) blood pressure (BP) of ⩽140/90 mmHg were required. Preexisting treatment-related toxicity must have recovered to grade 1 or better. Patients with brain metastases, cardiac arrhythmias requiring treatment, active infection, hemoptysis, recent thrombosis, or bleeding diatheses were excluded.

### Treatment plan

Three additional dose levels (DLs) incorporating b.i.d. oral S 5 days of 7 each week with every 2-week intravenous bevacizumab were tested as described in Table 1. There was a 6-week accrual pause between DLs to monitor for delayed toxicity. A history, blood pressure measurement, and urine protein/creatinine ratio were performed before each bevacizumab dose, with detailed history and physical examination every cycle (Azad *et al*, 2008b). If the urine protein/creatinine ratio was greater than 1.0 but less than grade 3, bevacizumab was given and a 24-h urine was collected for measurement of protein before the next cycle. Bevacizumab was held if proteinuria exceeded 2 g per 24 h.

### Dose-limiting toxicity and dose modifications

Dose-limiting toxicity (DLT) was defined as any recurrent grade 2 or single grade 3 or greater event related to study medications occurring within the first 6 weeks of treatment as delimited by the CTCAEv3 with the following exception: patients with a history of hypertension requiring therapy were allowed one additional antihypertensive drug, whereas up to two agents could be introduced to previously normotensive patients. Dose levels were expanded to six patients if a DLT was observed. Documented grade 2 or 3 toxicity required a hold in therapy until toxicity resolved to grade 1. Patients were dose reduced by one level for grade 3 toxicity or recurrent grade 2 toxicity; treatment was discontinued for grade 4 toxicity. Patients monitored BP at home daily for the first 4 weeks; BP in excess of 160/100 mmHg required intervention, with stability below that level for 3 days required for additional treatment. If two or more patients were found to have DLT, the MTD was considered to have been exceeded.

### Patient monitoring, response assessment, and statistics

Pretreatment assessments were performed within 2 weeks of therapy initiation. Patients were seen in clinic every 2 weeks for the first two cycles of treatment, then monthly. Reassessment imaging was performed every 8 weeks and evaluated by the reference radiologist without knowledge of the patient's clinical status. Tumour effects were characterised using RECIST v1.0 (Therasse *et al*, 2000).

## Results

### Patient accrual and dose escalation and determination of MTD

An additional 17 patients on DL 4 and 5 A/B were accrued and received a median of four cycles of therapy (range: 1.5–26 cycles). Two of five patients in DL 5B took 200 mg b.i.d. instead of 400 mg and were assessed as assigned (intent-to-treat). Nineteen patients with platinum-resistant EOC who received therapy on DLs 1–5 received a median of five cycles (range 1.5–37 cycles; Table 2). Dose-limiting toxicity was observed, defining DL 4 as the MTD (Table 3).

### Toxicity and dose modification

Dose-limiting toxicity of grade 3 hypertension and transaminitis was noted in DL 5A, and grade 3 diarrhoea, hypertension, and transaminitis were dose limiting in DL 5B (Table 4). Previously reported DLT was on DL 2, 200 mg b.i.d. continuously with 10 mg kg^−1^ bevacizumab every other week, and consisted of grade 3 thrombocytopaenia and proteinuria; these were not observed in DL 4 and 5 A/B. Additional serious adverse events on the intermittent schedule included two patients with cervical cancer on DL 5B who developed rectovaginal fistula (*n*=1, grade 2, after cycle 2) and appendiceal perforation (*n*=1, grade 3, after 4 cycles), respectively. Sorafenib was reduced to 200 mg daily, days 1–5 of 7 after fistula repair surgery. The patient with appendiceal perforation had sorafenib dose reduction to 400 mg once daily, days 1–5 of 7 for grade 2 HFSR and mucositis after cycle one. Both patients were treated with radiation therapy before study and had confirmed stable disease (SD) with this trial (7 and 5 months).

Overall, 7 of 17 (41%) patients required sorafenib reduction to 200 mg once daily, days 1–5 of 7. Dose modifications of sorafenib occurred at a median of 2 cycles for patients started on sorafenib 400 mg b.i.d. (DL 5B) and at a median 3.5 cycles for patients started on sorafenib 200 mg b.i.d. Primary cause for dose reduction was HFSR (*n*=5/7). Other causes were oral mucositis (*n*=2), anorexia/fatigue/weight loss (*n*=1), and fistula (*n*=1). Although the number of patients are small, we observed that fewer patients on DL 4–5 B required sorafenib dose reduction to 200 mg daily, than patients on the continuous sorafenib dose schedule (7/17=41% *vs* 29/39=74%). Eight of 10 patients who remained on treatment for 4+ months required sorafenib dose reduction.

Hypertension was an expected adverse event for both agents and we reported interactive increase in hypertension with the continuous schedule. Grade 1–3 hypertension developed in 76% (13 of 17) patients and required institution or modification of an antihypertensive regimen (Table 3). The protocol defined independent dose reduction criteria. The incidence of hypertension was similar between the two sorafenib dose schedules.

Skin rashes (14 HFSR and 2 other) were observed in 16 of 17 (94%) patients; sorafenib dose was reduced in six patients for recurrent grade 2 rashes (HFSR=5, ear rash=1). Grade ⩾2 mucositis occurred in five (29%) patients. One patient had mouth, tongue, throat, and anal mucositis. The other patient developed ear and perirectal desquamation and rashes. Temporary interruption of sorafenib administration for 3–5 days or reduction to a single dose of 200 mg daily was associated with a rapid symptom improvement. Although non-statistically significant, a trend towards less dermatologic toxicity with intermittent S was observed compared with original DLs (47 *vs* 59%).

Two study deaths occurred, one during a treatment hold and one shortly after treatment was discontinued. One patient in DL 5A with urothelial cancer died of progressive disease and pneumonia after cycle one. One patient in DL 5B with endometrial cancer developed a left leg deep vein thrombosis after cycle one, and died at home 1 week later. Drug had been held during initiation of anticoagulation and had not been reinstituted at the time of her death. Neither was felt to be probably or definitely related to drug.

### Clinical and tumour response

All patients enrolled into the study had progressive disease at the time of enrolment. Partial response or SD lasting ⩾4 months was seen in 10 of 17 (59%) patients on intermittent sorafenib treatment (Table 5), similar to the 59% rate observed in those receiving continuous sorafenib on DL 1–2. No loss of clinical benefit was thus apparent with the intermittent dosing of sorafenib.

### Analysis of all EOC patients receiving sorafenib and bevacizumab therapy

A further analysis of all EOC patients treated with bevacizumab and either continuous or intermittent sorafenib was undertaken. Six patients with platinum-resistant EOC were accrued in DL 4–5 B for a total of 19 patients over all DLs. The pattern of toxicity and dose reduction was not different in the EOC patients on continuous and intermittent sorafenib dosing. The most common causes of dose reduction in EOC patients were HFSR (*n*=8), anorexia/fatigue/weight loss (*n*=2), infection (*n*=2), other rash (*n*=2), thrombocytopaenia and proteinuria (*n*=1), and colon obstruction (*n*=1). Most (84%) experienced grade 1–3 hypertension. Grade 1–2 HFSR occurred in 18 (95%) of 19 EOC patients; grade 3 and 4 HFSR were not observed. No fistulae or perforations occurred in the six EOC patients on intermittent sorafenib. Clinical benefit was observed in 15 of 19 (79%) assessable patients (median, 8 months; range 4–37). These patients were heavily pretreated with cytotoxic agents, with a median of five previous treatments, and all had documented progressive disease before study. Confirmed PR was seen in 8 (42%) of 19 patients with EOC, including all DLs (20, 22, 37, 26, 13, 4, 26, and 8 months). Seven additional patients had disease stabilisation for at least 4 months. Our results show a promising response rate in heavily pretreated patients with platinum-resistant EOC with an acceptable tolerance of the refined sorafenib dose schedule.

## Discussion

Inhibition of angiogenesis has emerged as an important therapeutic strategy. We reported the first study of a combination therapy with two anti-VEGF-targeted agents applied in series. Our preliminary results showed an unexpectedly high response rate (46%) in patients with relapsed EOC (Sieczkiewicz *et al*, 2002; Jain, 2005). In the original dose escalation study, sorafenib at 200 mg b.i.d. with bevacizumab 5 mg kg^−1^ every 2 weeks was not tolerable long term and a substantial number of patients (62%) experienced fatigue, hand–foot syndrome, hypertension, proteinuria, and thrombocytopaenia. Although none of these side effects represents a new toxicity signal for these agents, the incidence of adverse events was greater than what would be expected through the use of each agent alone at standard doses. Therefore, we pursued this regimen with intermittent schedule using sorafenib 5 days of 7 each week. We observed a similar pattern of adverse events and a continued need to decrease the dose of sorafenib to 200 mg once daily, although not in as many patients as with the continuous daily dose schedule. Moreover, the patients did not lose clinical benefit after sorafenib reduction when given with bevacizumab. Eight of 10 patients who remained on the study for 4 months or more required sorafenib dose reduction (to 200 mg (*n*=6) or 400 mg (*n*=2) once daily, days 1–5 a week). Dose modification of sorafenib occurred on all three DLs. Hand–foot skin reaction was a common cause of dose reduction with both schedules. We also examined the potential value of this regimen in a group of 19 treated EOC patients and demonstrated value independent of the schedule and dose administered supporting the ongoing phase II trial.

A goal of the schedule of the components in this trial was to identify the optimal dose combination and the DLT. The pharmacodynamic interactions demonstrated that the tolerable dose of bevacizumab used is half or less of the recommended dose in other solid tumour studies (Azad *et al*, 2008b). We were unable to escalate beyond 5 mg kg^−1^ every 2 weeks because of proteinuria (*n*=2), thrombocytopaenia (*n*=1), and hypertension in DL 5A (*n*=2). Dose-limiting hypertension was less common with the intermittent sorafenib schedule. Thus, the intermittent sorafenib dose schedule in combination with bevacizumab is recommended for phase II application and is the dose and schedule of our ongoing phase II study in EOC.

Several phase I or II studies have investigated the combination of bevacizumab with other targeted agents. In a Phase I study of the combination of bevacizumab and sunitinib in 25 patients with metastatic renal cell cancer (mRCC), Feldman *et al* (2009) reported that the MTD was sunitinib 50 mg and bevacizumab 10 mg kg^−1^ every 2 weeks. Although a high objective response rate (52%) was observed, 48% discontinued study because of grade 3 or 4 hypertension, haematologic, or vascular toxicities. Another trial of this combination for all solid tumours is currently ongoing and has not observed the same high rates of adverse events. Hainsworth *et al* (2005) reported a 25% response rate and 1-year PFS of 43% in patients with mRCC treated with full-dose erlotinib and bevacizumab. Everolimus (10 mg daily), in combination with bevacizumab 10 mg kg^−1^ every 2 weeks, has been investigated in a phase II study in mRCC previously treated with sorafenib and/or sunitinib. Grade 3–4 proteinuria occurred in 19% with grade 1–2 toxicities of skin rash/pruritus (55%), mucositis/stomatitis (49%), and hypertension (25%). Objective response (21%) and SD (69%) were observed in 42 evaluable patients. Sosman and Puzanov (2009) evaluated the combination of sorafenib and bevacizumab in a phase I/II trial in mRCC yielding PRs in 4 of 14 patients. Hand–foot skin reaction, hypertension, and stomatitis were dose limiting. These phase I/II studies have provided preliminary evidence of potential clinical benefit of this combination of STIs with bevacizumab. The patterns of DLT vary depending upon the STI in the combination, in some cases, toxicity precluded further application of the combination. Optimising the management of adverse effects and better selection of patients for treatment with these combinations may improve the benefits.

The intermittent sorafenib dose schedule was based on the hypothesis that the 2-day drug holiday would be associated with a rapid reduction in symptoms. This was based on our observations identifying a rapid attenuation of severity of HFSR and constitutional symptoms; the pharmacokinetic profile of sorafenib is known to hit a stable steady state by 1 week. This schedule resulted in the same extent of clinical benefit as in the original DLs with continuous daily dosing of sorafenib (59%). Clinical benefit was observed in 59% of patients on our trial. Our patients were heavily pretreated, and all had documented progressive disease before study. Our results show a promising response rate in heavily pretreated patients with platinum-resistant EOC. Nine of 19 (47%) patients with EOC attained PR, and six patients had disease stabilisation for at least 4 months. Overall, clinical benefit was observed in 15 (79%) of 19 patients (median, 8 months; range, 4–37 months). These results compare favourably with the phase II results of single-agent bevacizumab (15 mg kg^−1^ every 3 weeks) in patients with minimally pretreated relapsed EOC, two or fewer earlier regimens, that yielded a 21% response rate, with 40% of patients progression-free at 6 months and no perforations (Burger *et al*, 2007). Cannistra *et al* (2007) reported a phase II trial of bevacizumab 15 mg kg^−1^ every 3 weeks in platinum-resistant EOC patients with three or fewer earlier regimens. In this study, response rate was 16%, median PFS 4.4 months and 11% of those enrolled experienced perforations. Although direct comparison of our trial is not feasible, we observed a higher response rate and duration of therapy among EOC patients, and our regimen had no perforations in 19 patients. On the basis of these promising phase I data, we are now conducting a phase II study of bevacizumab with this intermittent schedule of sorafenib in EOC.

## Figures and Tables

**Table 1:** Distribution of patients: dose levels (DLs; intent-to-treat)

**DL**	**No. (*n*=17)**	**Ovarian cancer (*n*=19)**	**Sorafenib (mg b.i.d., days every week)**		**Bevacizumab (mg kg^−1^ every 2 weeks)**	**Cycles, median (range)**
1	a	10	200	D1-7	5	9.5 (2–37)
2	a	3	200	D1-7	10	4 (4–5)
4^b^	7	3	200	D1-5	5	4 (2–26)
5A	5	2	200	D1-5	10	2 (2–9)
5B	5	1	400	D1-5	5	5 (2–8)

a Previously reported DL 1 (*n*=33) and DL 2 (*n*=6).

b DL 3 did not enroll because of dose-limiting toxicity at DL 2.

**Table 2:** Patient characteristics

	**DL 4, 5A, 5B (*n*=17)**	**Ovarian cancer (*n*=19^a^, all DLs)**
*Characteristics*	*Value*	
		
*Age, years*		
Median	60	52.5
Range	37–74 year old	40–61 year old
No. of patients	17	19
		
*ECOG performance status*		
0	5	1
1	12	18
		
*Sex*		
Female	14	19
Male	3	0
		
*Previous anticancer treatments*		
All, median (range)	3 (1–7)	5 (1–11)
Chemotherapy, median (range)	2 (1–7)	4 (1–9)^b^
Radiation, median (range)	1 (1–5)	0
		
*Tumour type*	*N*=17	
Ovarian cancer	6	19^a^
Uterine	3	
Cervical	2	
Breast	2	
Melanoma	1	
Sarcoma	1	
Basal cell cancer	1	
Urothelial cancer	1	

a Platinum resistant (*n*=19), sensitive (*n*=0).

b Dose levels (DL) 4, 5 A/B: hormonal therapy (*n*=1), immunotherapy (*n*=1).

Ovarian cancer: hormonal therapy (*n*=6), immunotherapy (*n*=2), targeted therapy (*n*=3).

**Table 3:** Grade 2–5 toxicity by maximum grade per patient (*N*=17)^a^

	**DL 4, 5A, 5B (*n*=17)**			**Ovarian cancer (*n*=19, all DLs)**		
	**Maximum toxicity grade (no. of patients)**			**Maximum toxicity grade (no. of patients)**		
	**G2**	**G3**	**G4**	**G2**	**G3**	**G4**
Diarrhoea	2	2	0	2	1	0
Fatigue	2	1	0	4	2	0
Fistula	1	0	0	2	0	0
Mucositis^a^	3	2	0	0	0	0
						
*Skin rashes*						
HFSR	8^a^	0	0	17	0	0
Other^b^	2	0	0	0	0	0
						
Hypertension	7	3	0	9	6	0
Perforation	0	1	0	0	0	0
Proteinuria	2	0	0	1	1	0
Thrombocytopaenia	0	0	0	0	1	0
Thrombosis	0	2	0	1	0	1
Transaminitis	2	2	0	3	1	0

DLs=dose levels; HFSR=hand–foot skin reaction.

a One patient had mouth, tongue, throat. and anal mucositis.

b Ear and perirectal desquamation and rashes.

**Table 4:** Toxicity comparison of intermittent *vs* continuous dose schedule

	**DL 4, 5A, 5B (*N*=17)**	**DL 1, 2 (*N*=39)**
	**Toxicity grade G2–4**	**No. of patients (%)**
Diarrhoea	4/17 (24)	7/39 (20)
Fatigue	3/17 (18)	15/39 (38)
Fistula	1/17 (6)	2/39 (5)
Mucositis^a^	5/17 (29)	NA
		
*Skin rashes*		
HFSR^b^	8^a^/17 (47)	23/39 (59)
Other^c^	2	
		
Hypertension	10/17 (59)	26/39 (67)
Perforation	1/17 (6)	1/39 (3)
Proteinuria	2/17 (12)	6/39 (15)
Thrombocytopaenia	0/17 (0)	2/39 (5)
Thrombosis	2/17 (12)	3/39 (8)
Transaminitis	4/17 (24)	13/39 (33)

DLs=dose levels; HFSR=hand–foot skin reaction; NA=not applicable.

a One patient had mouth, tongue, throat, and anal mucositis.

b Although the numbers are small, when the difference in HFSR is compared between those receiving intermittent and continuous sorafenib schedules using the *χ*^2^-test, *P*=0.08.

c Ear and perirectal desquamation and rashes (separate patient with HFSR).

**Table 5:** Clinical outcome

**Dose level**	***n***	**Best response**	**Time on study (months)**
1	8^a^	**PR** **(5)**	**22, 26, 13**, **37, 20**
		SD (3)	6, 5, 4
2	3^a^	**PR** **(1)**	**4**
		SD (2)	5, 4
4	2^a^	**PR** **(1)**	**26**
		SD (1)	5
	1(uterine)	SD (1)	4
	1(br)	SD (1)	4
	1(bcc)	SD (1)	4
5a	1^a^	SD (1)	8
	1	SD (1)	9
5b	1^a^	**PR** **(1)**	**8**
	2 (cx)	SD (2)	5, 7

**All ovarian cancer (*N*=19; all DLs)**	**Intermittent sorafenib (DL 4, 5A, 5B; *N*=17)**
PR 8/19=42%	PR 2/17 (12%)
PR or SD⩾ 4 cycles; 15/19=79%	PR or SD⩾ 4 cycles; 10/17 (59%)

cx=cervical cancer; bcc=basal cell cancer; br=breast cancer; PR=partial response; SD=stable disease;

a ovarian cancer.

SD other (1 each): melanoma, sarcoma, urothelial cancer.
